# Brimonidine prevents neurodegeneration in a mouse model of normal tension glaucoma

**DOI:** 10.1038/cddis.2014.306

**Published:** 2014-07-17

**Authors:** K Semba, K Namekata, A Kimura, C Harada, Y Mitamura, T Harada

**Affiliations:** 1 Visual Research Project, Tokyo Metropolitan Institute of Medical Science, Tokyo, Japan; 2 Department of Ophthalmology, Institute of Health Biosciences, The University of Tokushima Graduate School, Tokushima, Japan

## Abstract

Glaucoma is one of the leading causes of irreversible blindness that is characterized by progressive degeneration of optic nerves and retinal ganglion cells (RGCs). In the mammalian retina, excitatory amino-acid carrier 1 (EAAC1) is expressed in neural cells, including RGCs, and the loss of EAAC1 leads to RGC degeneration without elevated intraocular pressure (IOP). Brimonidine (BMD) is an *α*2-adrenergic receptor agonist and it is commonly used in a form of eye drops to lower IOP in glaucoma patients. Recent studies have suggested that BMD has direct protective effects on RGCs involving IOP-independent mechanisms, but it is still controversial. In the present study, we examined the effects of BMD in EAAC1-deficient (KO) mice, an animal model of normal tension glaucoma. BMD caused a small decrease in IOP, but sequential *in vivo* retinal imaging and electrophysiological analysis revealed that treatment with BMD was highly effective for RGC protection in EAAC1 KO mice. BMD suppressed the phosphorylation of the *N*-methyl-D-aspartate receptor 2B (NR2B) subunit in RGCs in EAAC1 KO mice. Furthermore, in cultured Müller glia, BMD stimulated the production of several neurotrophic factors that enhance RGC survival. These results suggest that, in addition to lowering IOP, BMD prevents glaucomatous retinal degeneration by stimulating multiple pathways including glia–neuron interactions.

Glaucoma is one of the leading causes of vision loss in the world. It is estimated that glaucoma will affect more than 80 million individuals worldwide by 2020, with at least 6–8 million individuals becoming bilaterally blind.^1^ The disease is characterized by the progressive degeneration of retinal ganglion cells (RGCs) and their axons, which are usually associated with elevated intraocular pressure (IOP). On the other hand, normal tension glaucoma (NTG) is a subtype of glaucoma that presents with statistically normal IOP. The prevalence of NTG is reported to be higher among the Japanese than among Caucasians.^2^ These findings suggest a possibility that non-IOP-dependent factors may contribute to disease progression of glaucoma, especially in the context of NTG.^3, 4^ For example, an excessively high extracellular concentration of glutamate chronically activates glutamate receptors, such as *N*-methyl-D-aspartate (NMDA) receptors, and allows calcium entry into the cell causing an uncontrolled elevation of intracellular calcium levels. This process is thought to be one of the causes of RGC death.^3, 4, 5^ The glutamate transporter (GLT) is the only mechanism for removal of glutamate from the extracellular fluid in the retina.^3, 6, 7^ In the inner plexiform layer where synapses exist across RGCs, at least three transporters are involved in this task: GLT-1 located in the bipolar cell terminals; excitatory amino-acid carrier 1 (EAAC1) in RGCs; and glutamate/aspartate transporter (GLAST) in Müller glial cells.^3, 7, 8^ We previously reported that EAAC1 and GLAST knockout (KO) mice show progressive RGC loss and optic nerve degeneration without elevated IOP, and not only glutamate neurotoxicity but also oxidative stress is involved in its mechanism.^3, 8, 9, 10^ In adult EAAC1 and GLAST KO mice, lipid hydroperoxides were increased and glutathione concentrations were decreased in retinas, suggesting the involvement of oxidative stress in RGC loss. In addition, cultured RGCs prepared from EAAC1 KO mice were more vulnerable to oxidative stress.^3^ Oxidative stress has been proposed to contribute to retinal damage in various eye diseases including glaucoma and age-related macular degeneration.^11, 12^ Taken together with the downregulation of GLTs and glutathione levels observed in glaucoma patients,^13^ these mice seem to be useful as the animal models of NTG.

Brimonidine (BMD) is a selective *α*2-adrenergic receptor agonist that lowers IOP by reducing the production of aqueous humor and facilitating its exit via the trabecular meshwork.^14^ Recent studies have shown that BMD protects RGCs from glutamate neurotoxicity, oxidative stress and hypoxia *in vitro*.^15, 16^ In addition, BMD provides neuroprotective effects in various animal models of optic neuropathy including experimental glaucoma, ischemia, oxidative stress and optic nerve injury.^17, 18, 19^ BMD may exert its neuroprotective effects via the upregulation of neurotrophic factors, such as brain-derived neurotrophic factor (BDNF)^20^ and basic fibroblast growth factor (bFGF),^21, 22^ in RGCs. Thus, the neuroprotective effects of BMD seem to be, at least partly, through IOP-independent factors, but the detailed mechanism are still unknown. Fujita *et al.*^23^ recently reported that topical administration of BMD promotes axon regeneration after optic nerve injury. BMD increased the expression of the tropomyosin receptor kinase B (TrkB), a high-affinity BDNF receptor, in the mouse retina. We previously reported that BDNF-TrkB signaling in Müller glial cells have important roles in the production of trophic factors including BDNF and bFGF, and in the protection from glutamate-induced RGC death and drug-induced photoreceptor death.^24^ Systemically administered *α*2-adrenergic agonists are known to activate selectively extracellular signal-regulated kinases in Müller cells *in vivo*.^25^ These results suggest a possibility that BMD may stimulate the production of trophic factors in not only RGCs but also in Müller cells. In the present study, we show that BMD prevents glaucomatous retinal degeneration in EAAC1 KO mice, an animal model of NTG, and we report novel IOP-independent pathways for BMD-mediated neuroprotection that involve NMDA receptors and glia–neuron interaction.

## Results

### BMD protects RGCs in EAAC1 KO mice

**Figure 1:**
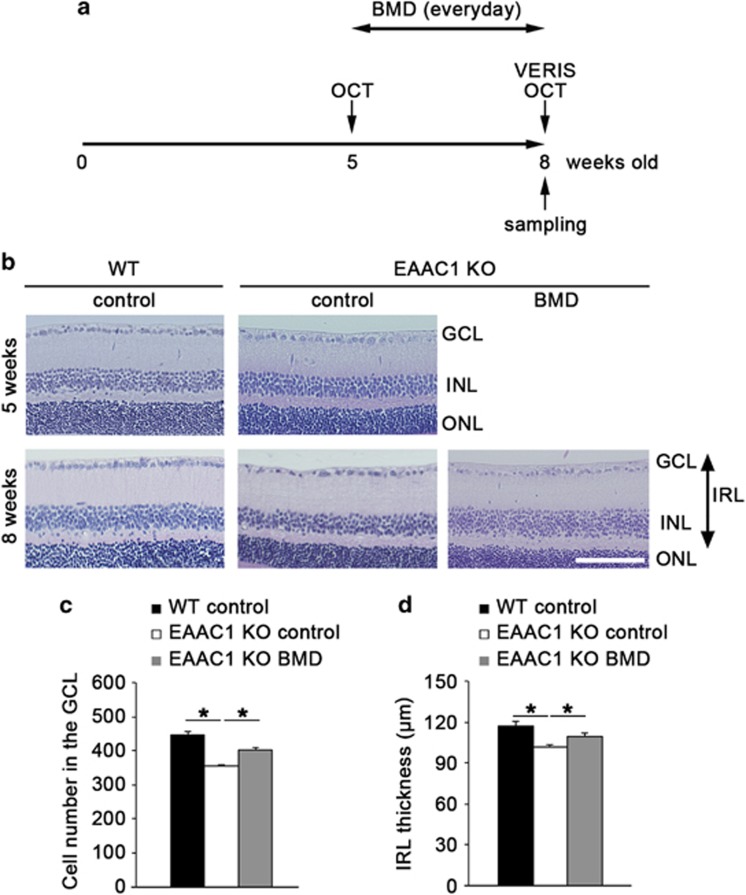
Effects of BMD on RGC loss and IRL thickness in EAAC1 KO mice. (**a**) Animal protocols. 1.0% BMD or PBS (5 *μ*l) was administered locally every day from 5W to 8W. The mice were killed at 5W and 8W. (**b**) H&E staining of retinal sections at 5W and 8W in WT and EAAC1 KO mice. Scale bar: 100 *μ*m. INL, inner nuclear layer; . (**c** and **d**) Quantification of the RGC number (**c**) and IRL thickness (**d**). The data are presented as means±S.E.M. of six samples for each experiment. *P<0.05

**Figure 2:**
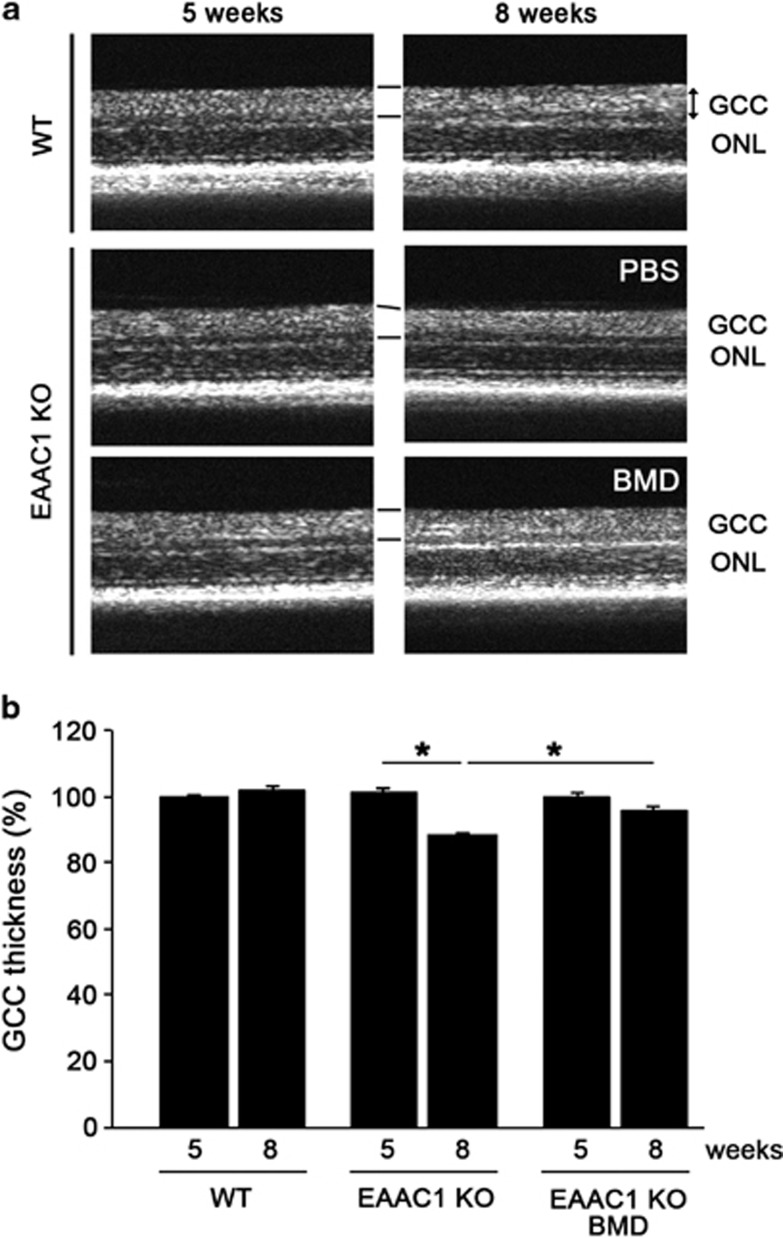
*In vivo* imaging of the retina in EAAC1 KO mice treated with BMD. (**a**) OCT cross-sectional images of retinas at 5W and 8W. (**b**) Longitudinal evaluation of the GCC thickness. The data are presented as means±S.E.M. of six samples for each experiment. *P<0.05

To investigate whether BMD is capable of preventing the NTG-like phenotypes in EAAC1 KO mice, we administered BMD or PBS (control) locally every day to EAAC1 KO mice from 5 weeks old (5W) to 8W (Figure 1a). The retinas of EAAC1 KO mice show normal organization at 5W, but RGC loss and the thinning in the inner retina was clear at 8W (Figure 1b).^3, 8, 10^ The cell number in the ganglion cell layer (GCL) at 8W was significantly lower in EAAC1 KO mice (356±3; *n*=6) compared with WT mice (448±8; *n*=6) (Figure 1c). In addition, the thickness of the inner retinal layer (IRL) was significantly decreased at 8W in EAAC1 KO mice (100±2 *μ*m; *n*=6) (Figure 1d). In BMD-treated EAAC1 KO mice, the number of surviving neurons was significantly higher (396±3; *n*=6) than that in control EAAC1 KO mice (Figures 1b and c). In addition, BMD treatment prevented the thinning of the IRL (108±2 *μ*m; *n*=6) (Figure 1d). We also visualized retinal layers using optical coherence tomography (OCT), a noninvasive imaging technique that can be used to acquire cross-sectional tomographic images of the retina *in vivo*.^10, 26^ The average thickness of the ganglion cell complex (GCC), which includes the nerve fiber layer, GCL, and inner plexiform layer, was significantly greater in BMD-treated EAAC1 KO mice (99±1% *n*=6) compared with control EAAC1 KO mice (88±1% *n*=6) (Figure 2). These data indicate that BMD treatment protects RGCs from NTG-like neurodegeneration.

### BMD improves visual function in EAAC1 KO mice

**Figure 3:**
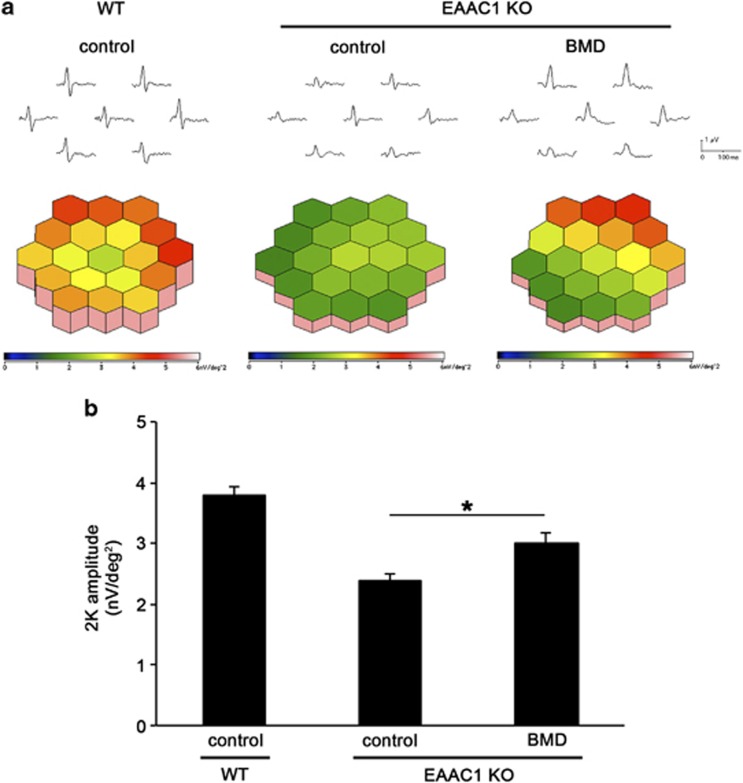
Effects of BMD on visual responses in EAAC1 KO mice. (**a**) Averaged visual responses of the second-order kernel (2K) demonstrated using 3D plots. (**b**) Quantitative analysis of the visual response amplitude. The data are presented as means±S.E.M. of six samples for each experiment. *P<0.05

To determine whether the observed BMD-induced RGC protection is functionally significant, we analyzed multifocal electroretinograms (mfERGs) of EAAC1 KO mouse retinas. Functional RGCs contribute to eliciting the mfERG response, and the second-order kernel (2K), which appears to be a sensitive indicator of inner retinal dysfunction,^27^ is impaired in glaucoma patients.^28^ The averaged 2K responses of six mice are shown in Figure 3a. The response topography demonstrated that the 2K component derived from EAAC1 KO mice tended to be smaller in all visual fields compared with that from WT mice. Quantitative analysis revealed that the 2K amplitude was reduced in EAAC1 KO mice (2.4±0.1 nV/deg^2^; *n*=6) compared with that in WT mice (3.8±0.1 nV/deg^2^; *n*=6) (Figure 3b). However, BMD prevented the reduction of the 2K amplitude in EAAC1 KO mice (3.0±0.2 nV/deg^2^; *n*=6), suggesting that the neuroprotective effect of BMD is functionally significant in EAAC1 KO mice.

### Effects of BMD on IOP in EAAC1 KO mice

**Figure 4:**
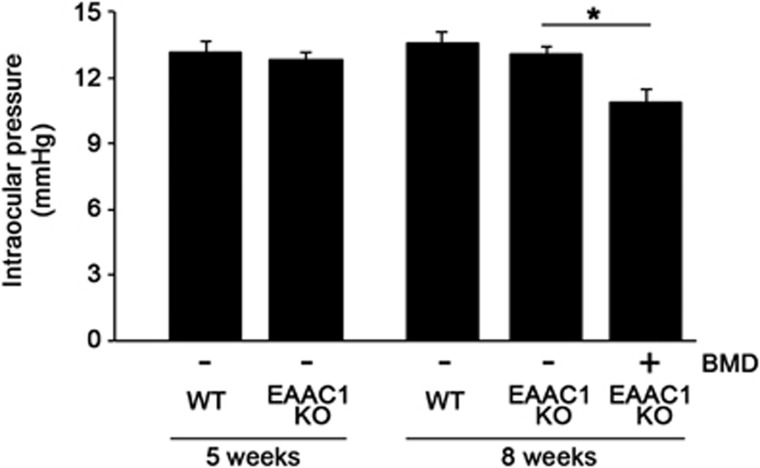
Effects of BMD on IOP in EAAC1 KO mice. The data are presented as means±S.E.M. of six samples for each experiment. *P<0.05

IOP measurement was carried out at 5W and 8W mice at around 2100 hours, when IOP is the highest in mouse eyes.^29^ The IOP of EAAC1 KO mice was not significantly increased compared with WT at both 5W and 8W (Figure 4). BMD slightly, but significantly, reduced IOP (10.8±0.6 mmHg; *n*=6) compared with control EAAC1 KO mice (13.1±0.3 mmHg; *n*=6). These results suggest that BMD-induced RGC protection may, at least partly, involve an IOP-dependent mechanism.

### BMD reduces phosphorylated NR2B expression in EAAC1 KO mice

**Figure 5:**
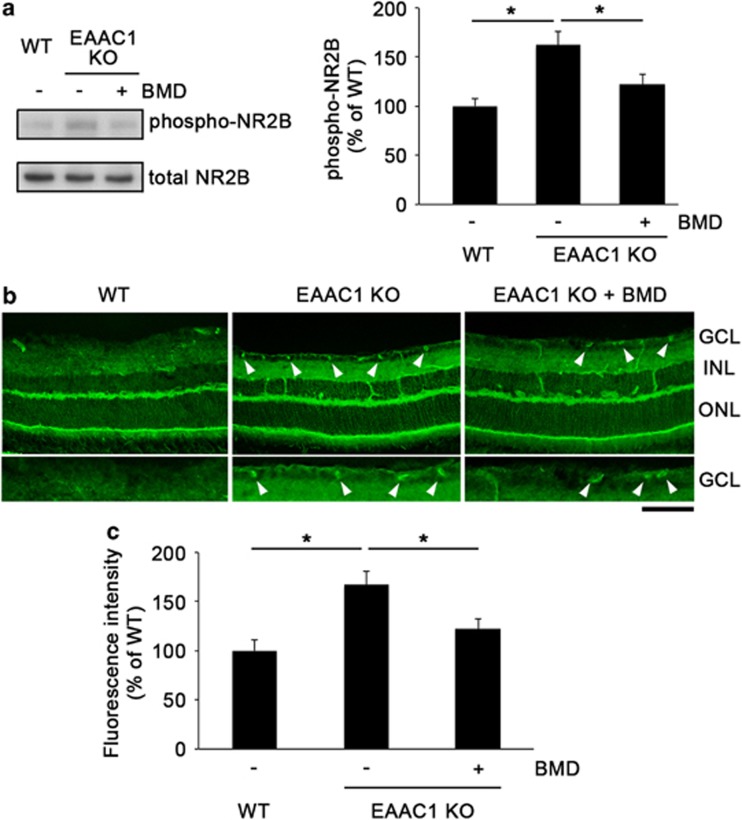
Effects of BMD on phosphorylation of NR2B in the retina. (**a**) Immunoblot analysis of phosphorylated and total NR2B in the retinas of WT, EAAC1 KO and BMD-treated EAAC1 KO mice. Ratio of NR2B phosphorylation in WT mice was estimated as 100%. (**b**) Immunohistochemical analysis of mouse retinas stained with an antibody against phosphorylated NR2B in WT, EAAC1 KO and BMD-treated EAAC1 KO mice. Arrowheads indicate RGCs. INL, inner nuclear layer. Scale bar: 100 and 50 *μ*m in upper and lower rows, respectively. (**c**) Quantitative analysis of the phosphorylated NR2B intensity in (**b**). The NR2B fluorescence intensity in WT mice was estimated as 100%. The data are presented as means±S.E.M. of six samples for each experiment. *P<0.05

We recently reported that inactivation of NR2B, one of the NMDA receptor subunits, protects RGCs from glutamate neurotoxicity.^5^ Since BMD exerts direct neuroprotective effects on RGCs,^15, 16^ BMD may affect the NMDA receptor activity in the retina. To determine this possibility, we next examined total and phosphorylated (at Tyr1472) NR2B protein expressions in EAAC1 KO mice before and after BMD treatment. The ratio of phosphorylated/total NR2B in EAAC1 KO mice was significantly higher compared with that in WT mice (161±13% *n*=6), but such an increased level was not detected after BMD treatment (123±12% *n*=6) (Figure 5a). Immunohistochemical analysis revealed that immunoreactivity of phosphorylated NR2B was low in WT mice (100±11% *n*=6), but it was significantly increased, especially in RGCs, in EAAC1 KO mice (167±14% *n*=6) at 8W (Figures 5b and c), suggesting an excitotoxic environment in EAAC1 KO mice. The elevation of the phosphorylated NR2B expression was significantly suppressed by BMD treatment (122±10% *n*=6). These results suggest that BMD exerts a direct neuroprotective effect, at least partly, by reducing the activated NMDA receptor expression and thus suppressing excitotoxicity.^15, 16, 17, 18^

### BMD stimulates trophic factor productions in Müller glial cells

**Figure 6:**
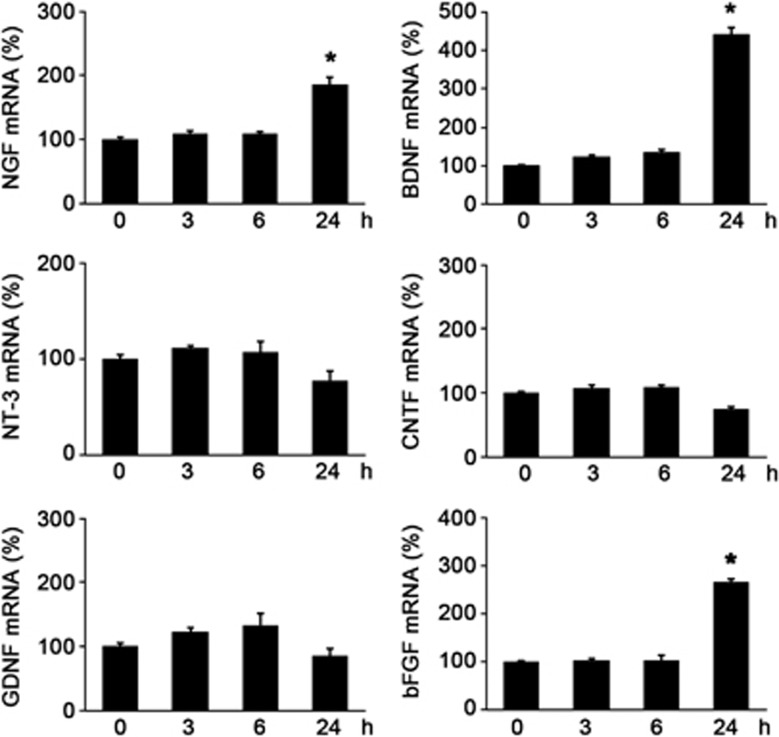
Effects of BMD on trophic factor productions in cultured Müller cells. mRNA expression levels of NGF, BDNF, NT-3, CNTF, GDNF and bFGF were determined using quantitative real-time PCR. Glyceraldehyde 3-phosphate dehydrogenase (GAPDH) was used as an internal control. Each mRNA production level before stimulation (0 h) was estimated as 100%. The data are presented as means±S.E.M. of six samples for each experiment. *P<0.01

The cross-talk between glia and neurons in neuroprotection is now well known, and during retinal degeneration, Müller glial cells produce several trophic factors and rescue retinal neurons.^30, 31^ Thus, we next examined whether BMD stimulates trophic factor production in cultured Müller cells prepared from WT mice. BMD significantly increased mRNA expression levels of the nerve growth factor (NGF; 186±12% *n*=6), BDNF (441±18% *n*=6) and bFGF (265±8% *n*=6), but not of neurotrophin-3 (NT-3), ciliary neurotrophic factor (CNTF) and glial cell line-derived neurotrophic factor (GDNF) (Figure 6). These results suggest that BMD has the capacity to stimulate the production of several trophic factors in Müller cells, and these trophic factors may act on RGCs leading to RGC protection indirectly.^24^

## Discussion

This was the first study to investigate the neuroprotective effects of BMD in a mouse model of NTG. We showed that BMD prevents progressive RGC loss, thinning of the IRL and visual impairment in EAAC1 KO mice. To demonstrate these findings in the same animal, we used OCT and mfERG that permit *in vivo*, noninvasive, longitudinal and quantitative assessment of the changes in retinal morphology and function in EAAC1 KO mice. These techniques clearly visualized the neuroprotective effects of BMD in the present study and they are useful for providing information in experimental animals as well as in clinical trials and management.^32^ The IOP of EAAC1 KO mice are low and BMD slightly decreased the IOP of these mice, suggesting that the clear neuroprotective effects of BMD are mostly independent of its ability to lower IOP. This concept supports the findings from a recent study that reported low-pressure glaucoma patients treated with BMD 0.2% are less likely to have field progression than patients treated with timolol 0.5%.^33^ As the mean IOP was similar in both groups at the 4-year follow-up, these results suggested that BMD has IOP-independent neuroprotective effects on RGCs. Our data suggest that one of the IOP-independent mechanisms is suppression of excitotoxicity in RGCs. Excitotoxicity is implicated in the degeneration of RGCs and optic nerves observed under pathologic conditions including glaucoma.^5, 34^ In addition, overactivation of NMDA receptors is thought to be a key contributing factor in the pathophysiology of many central nervous system disorders, such as Alzheimer's disease^35^ and Huntington disease.^36^ In the EAAC1 KO mouse retina, the phosphorylated NR2B expression level was upregulated, and BMD significantly inhibited this effect, suggesting that excitotoxicity is reduced in BMD-treated EAAC1 KO mice leading to increased RGC survival. Another IOP-independent mechanism may be stimulation of glia–neuron interactions. Previous studies have shown that BMD directly increases BDNF and bFGF production in RGCs.^15, 16, 21, 22^ However, production of neurotrophic factors in Müller glial cells has not been investigated previously. We found that BMD stimulates the production of NGF, BDNF and bFGF, all of which induce RGC survival, in Müller glial cells.^24, 37^ Taken together, in addition to lowering IOP, BMD may prevent glaucomatous retinal degeneration by stimulating multiple pathways including glia–neuron interactions.

Recent studies have reported a possibility that blockade of axonal transport in glaucoma leads to deficits in the neurotrophic factors and subsequent RGC death in adult eyes.^38^ Retrograde transport of radiolabeled BDNF was impaired following IOP increase in rats, and accumulation of TrkB immunolabeling was found in the optic nerve head in this model.^39^ Therefore, supplementation therapy using neurotrophic factors is an attractive method for protection of RGCs and axons in glaucoma. We previously proposed a model in which exogenously applied or microglia-derived neurotrophins regulate photoreceptor survival indirectly by regulating secondary trophic factor production in Müller cells.^31^ However, the effects that such a glia–neuron interaction would have *in vivo* or whether it would be relevant for the protection of neural cell types that express TrkB remained unclear. To explore this point, we prepared two conditional KO mice in which TrkB was deleted from Müller glia (TrkB GFAP KO mice) or from two types of retinal neurons (RGCs and amacrine cells).^24^ Surprisingly, the extent of glutamate-induced retinal degeneration was similar in these two mutant mice. Furthermore, BDNF failed to increase the production of BDNF, bFGF, CNTF and GDNF in cultured Müller cells prepared from TrkB GFAP KO mice. These results suggest a possibility that BMD-induced activation of BDNF signaling in glia is critically involved in RGC protection. One interesting point is that BMD increased NGF production in Müller cells. A recent study reported that NGF eye drops reduced RGC loss in patients with advanced glaucoma, and indicated long-lasting improvements in visual field, optic nerve function, contrast sensitivity and visual acuity.^40^ However, several studies have failed to observe NGF-mediated RGC survival,^41, 42^ thus further studies are required before topical NGF can be considered for application in human glaucoma. Based on our findings, IOP-independent neuroprotection by BMD may be partly explained by BMD-induced upregulation of trophic factors in surrounding glial cells. However, another important point to consider is that Müller cells may exert both neuroprotective and detrimental effects during retinal degeneration.^10, 26, 30, 31, 37^ In animal models of high IOP, BMD treatment suppressed the GFAP immunoreactivity compared with the animals after treatment with timolol.^43, 44^ The mechanism by which BMD influences GFAP immunoreactivity is not well understood, but BMD may afford greater neuroprotection by attenuating the retinal glial reaction. We cannot directly compare these results with our present results from NTG model mice, but future studies examining the effects of gliosis on trophic factor productions in Müller cells may be interesting.

In summary, we showed that BMD prevents RGC death and reduces a degree of visual impairment in a mouse model of NTG by suppressing excitotoxicity in RGCs and stimulating trophic factor release from Müller cells, indicating that BMD is capable of activating several pathways in both neural and glial cells. There has been considerable progress in characterization of molecular pathways that regulate RGC survival. To this end, therapeutic potentials of several neurotrophic factors in protecting RGCs are emerging and some clinical trials for retinal diseases are underway.^45, 46^ Therefore, it is important to establish the long-term effects of BMD, and to determine whether BMD can synergistically stimulate RGC survival in combination with currently available therapy in glaucomatous eyes.

## Materials and Methods

### Mice

Experiments were performed using EAAC1 KO mice (Miltenyi Biotec GmbH, Bergisch Gladbach, Germany)^3, 8, 10^ in accordance with the Tokyo Metropolitan Institute of Medical Science Guidelines for the Care and Use of Animals.

### Drug administration

Mice were treated with 5 *μ*l drops of 1.0% BMD tartrate (Senju Pharmaceutical Co., Ltd, Osaka, Japan) dissolved in PBS every day from 5W to 8W. PBS was administered as a control.

### Histologic and morphometric studies

Paraffin-embedded retinal sections of 7 *μ*m thickness were cut through the optic nerve and stained with hematoxylin and eosin (H&E). The RGC number and the extent of retinal degeneration were quantified in two ways.^9^ First, the thickness of the IRL (between the internal limiting membrane and the interface of the outer plexiform layer and the outer nuclear layer) was analyzed. Second, in the same sections, the number of neurons in the GCL was counted from one ora serrata through the optic nerve to the other ora serrata.

### Imaging acquisition of spectral-domain OCT

Spectral-domain OCT (SD-OCT) (RS-3000; Nidek, Aichi, Japan) examinations were performed at 5W and 8W. For fundus imaging, polymethyl methacrylate contact lenses optimal for mice (UNICON, Osaka, Japan) were placed on the corneas. Use of the lenses prevents anesthesia-induced cataract progression. A 60-D adaptor lens was placed on the objective lens of the Multiline OCT to focus on the mouse retina. All the images were location matched, scanning vertically through the center of the optic nerve head at 3 disc diameter lengths above the optic nerve head.^10, 26^ The average thickness of GCC (between the internal limiting membrane and the interface of the inner plexiform layer and the inner nuclear layer) was measured. In this study, the maximum number of B-scans set by the manufacturer (50 for line scans) was used for averaging.

### mfERG

Mice at 5W and 8W were anesthetized by intraperitoneal injection of sodium pentobarbital. The pupils were dilated with 0.5% phenylephrine hydrochloride and 0.5% tropicamide. mfERGs were recorded using a VERIS 6.0 system (Electro-Diagnostic Imaging, Redwood City, CA, USA). The visual stimulus consisted of seven hexagonal areas scaled with eccentricity. The stimulus array was displayed on a high-resolution black and white monitor driven at a frame rate of 100 Hz. The second-order kernel, which is impaired in patients with glaucoma, was analyzed.^3, 9, 28^

### IOP measurement

IOP was measured by a commercial rebound tonometer (TonoLab; Colonial Medical Supply, Franconia, NH, USA) in anesthetized mice as reported previously.^9, 10^ To minimize variation, the data were collected during a time window of 4–6 min after injection of the anesthetic, during which IOP plateaus. IOP was measured at 5W and 8W. As the 24 h IOP pattern in mouse eyes is biphasic, with IOP being the highest at around 2100 hours,^29^ we examined IOP between 2000 and 2300 hours.

### Immunoblot analysis

Immunoblotting was performed as reported previously.^5^ Membranes were incubated with an antibody against NR2B (1 : 1000; BD Biosciences, San Jose, CA, USA), or phosphorylated NR2B (1 : 300; BD Biosciences) or actin (1 : 1000; BD Biosciences).

### Immunohistochemistry

Retinas were examined by immunostaining as reported previously.^5, 9, 10^ Immunohistochemistry was performed using the antibody for phosphorylated NR2B (1 : 1000; BD Biosciences). Quantitative analysis of the stained region was carried out using BZ-H1C (Keyence Software, Osaka, Japan).

### Cell culture and quantitative real-time PCR

Primary Müller cells^10, 24, 30, 31^ derived from WT mice were stimulated with BMD (1 *μ*M) for 3, 6, 12 and 24 h, and processed for quantitative PCR analysis. Quantitative RT-PCR was performed using the ABI 7300 Real-Time PCR system (Applied Biosystems, Foster City, CA, USA) with Power SYBR Green PCR Master Mix (Applied Biosystems) as reported previously.^47^ Complementary DNA reverse-transcribed from total RNA was amplified using primers specific for NGF (sense: 5′-CGACTCCAAACACTGGAACTCA-3′ antisense: 5′-GCCTGCTTCTCATCTGTTGTCA-3′), BDNF (sense: 5′-ATGCCGCAAACATGTCTATGAG-3′ antisense: 5′-TGACCCACTCGCTAATACTGTCA-3′), NT-3 (sense: 5′-GTTCCAGCCAATGATTGCAA-3′ antisense: 5′-GGGCGAATTGTAGCGTCTCT-3′), CNTF (sense: 5′-GGTGACTTCCATCAGGCAATACA-3′ antisense: 5′-CTGTTCCAGAAGCGCCATTAAC-3′), GDNF (sense; 5′-GGCCTACCTTGTCACTTGTTAGC-3′ antisense: 5′-GGCCTACTTTGTCACTTGTTAGC-3′), bFGF (sense: 5′-CACCAGGCCACTTCAAGGA-3′ antisense: 5′-GATGGATGCGCAGGAAGAA-3′) and GAPDH (sense: 5′-TGCACCACCAACTGCTTAG-3′ antisense: 5′-GGATGCAGGGATGATGTTC-3′).

### Statistics

For statistical comparison of two samples, we used a two-tailed Student's *t*-test. Data are presented as means±S.E.M. *P*<0.05 was regarded as statistically significant.

## Abbreviations

BDNF: brain-derived neurotrophic factor
bFGF: basic fibroblast growth factor
BMD: brimonidine
EAAC1: excitatory amino-acid carrier 1
GCL: ganglion cell layer
GFAP: glial fibrillary acidic protein
GLAST: glutamate/aspartate transporter
IOP: intraocular pressure
mfERG: multifocal electroretinogram
NMDA: *N*-methyl-D-aspartate
OCT: optical coherence tomography
RGC: retinal ganglion cell
